# Transdermal delivery of Minoxidil using HA-PLGA nanoparticles for the treatment in alopecia

**DOI:** 10.1186/s40824-019-0164-z

**Published:** 2019-10-31

**Authors:** Woo Yeup Jeong, Sodam Kim, So Yun Lee, Hyeseon Lee, Dong Wook Han, Seung Yun Yang, Ki Su Kim

**Affiliations:** 1 Department of Organic Materials Science and Engineering, College of Engineering, Pusan National University, 2 Busandaehak-ro 63 beon-gil, Geumjeong-gu, Busan, 46241 Republic of Korea; 2 Department of Biomaterials Science, Life and Industry Convergence Institute, Pusan National University, 1268-50 Samrangjin-ro, Miryang, 50463 Republic of Korea; 3 Department of Optic-Mechatronics Engineering, College of Nanoscience and Nanotechnology, Pusan National University, 2 Busandaehak-ro 63 beon-gil, Geumjeong-gu, Busan, 46241 Republic of Korea

**Keywords:** Hyaluronate, Poly(Lactide-co-Glycolide), Alopecia, Minoxidil, Transdermal drug delivery

## Abstract

**Background:**

Alopecia has become a very common disease that many people around the world are suffered. Minoxidil (MXD) is the most well-known commercialized drug in its treatment. However, in the case of MXD administration, there are some problems with low efficiency of transdermal delivery and additional side effects.

**Method:**

MXD and Rhodamine B (Rho B) are encapsulated in poly(Lactide-co-Glycolide) grafted hyaluronate nanoparticles (HA-PLGA/MXD NPs, HA-PLGA/Rho B NPs) which is prepared with W/O/W solvent evaporation method. After then, the investigation is carried out to confirm the feasibility of NPs in alopecia treatment.

**Results:**

Both of HA-PLGA/MXD NPs and HA-PLGA/Rho B NPs are successfully prepared. In addition, it is confirmed that HA-PLGA NPs sufficiently delivered to cells without any significant cytotoxicity by cell viability, cellular uptake and skin permeation test.

**Conclusion:**

Taken together, HA-PLGA NPs as a transdermal delivery carrier to hair follicle cells can be exploited to develop the efficient and effective platform of transdermal drug delivery for the treatment of various diseases.

## Introduction

Alopecia is a symptom that hairs were abnormally lost because of various causes including genetic disorder, healthy state, stress, and aging [1, 2]. Alopecia has become a common disease that large number of people around the world have suffered [3]. Many kinds of medical trial have been performed to treat alopecia by activating hair regeneration at follicle cells. Among them, medication is the most common method to treat alopecia.

Minoxidil (MXD) is the most widely used drug in alopecia treatment, especially male-pattern hair loss [1–7]. It is known to have a vasodilatory effect and expedite secretion of growth factors for hair regeneration. Due to the characteristics of alopecia, it works effectively when applied to the topical skin area through transdermal delivery of MXD. However, typical MXD delivery has the disadvantages of low efficiency of transdermal delivery and various side effects, such as tolerance, inflammation and low blood pressure [6]. Therefore, many studies have been investigated to improve the transdermal delivery efficiency of MXD applied to the skin and the therapeutic efficacy in alopecia, through the transdermal routes such as hair follicles, sweat glands or microneedle arrays [2, 4, 7–10].

To resolve the above issues, we have developed the transdermal drug delivery platform for the treatment of alopecia using MXD encapsulated Hyaluronate – Poly(Lactide-*co*-Glycolide) nanoparticles (HA-PLGA/MXD NPs). Hyaluronic acid (HA) is a well-known natural polysaccharide for various biomedical applications [11–17]. The excellent characteristic of HA for the transdermal delivery has been reported in our previous works [12]. Poly(Lactide-*co*-Glycolide) (PLGA) is also well-known biocompatible polymers and widely investigated for the various drug delivery application [11, 13, 18, 19]. Because of the hydrophobicity of PLGA, it is known to be permeable to the skin, and transdermal delivery using PLGA has been reported in some previous studies [15, 16]. Therefore, through the respective transdermal characteristics, HA-PLGA is expected to exhibit the synergistic effect in transdermal delivery of MXD. In this work, to encapsulate hydrophilic MXD in hydrophobic HA-PLGA conjugates we prepared NPs using the W/O/W emulsion method and characterized HA-PLGA/MXD NPs. Then we investigated cell viability, cellular uptakes and transdermal delivery of those NPs to confirm the enhanced transdermal delivery of MXD and the feasibility for treatment of various diseases on skin.

## Methods

### Materials

Hyaluronic acid (HA, 100 kDa) was purchased from Lifecore Co. (Chaska, MN). Poly(Lactide-co-Glycolide) (PLGA, 75:25, MW 10 kDa) was obtained from Wako Pure Chemical Industries, Ltd. (Osaka, Japan). Hexamethylenediamine (HMDA), N-Hydroxysuccinimide (NHS), Rhodamine B (Rho B), Poly(vinyl alcohol) (PVA), Dichloromethane (DCM), Tween 80, Formaldehyde were purchased from Sigma-Aldrich (St. Louis, MO). N,N′-Dicyclohexylcarbodiimide (DCC), Dimethylsulfoxide (DMSO), Ethanol, and Methanol were obtained from Alfa Aesar (Haverhill, MA). 1-Ethyl3-(3-dimethylaminopropyl) carbodiimide (EDC) hydrochloride, Minoxidil (MXD) was purchased from Tokyo Chemical Industry Co. (Tokyo, Japan). EZ-CYTOX was purchased from Daeil lab Service Co. (Cheongwon, Korea), Optimal cutting temperature (OCT) compound was obtained from Sakura Finetek (Zoeterwoude, Netherlands). Phosphate buffered saline (PBS) were purchased from Invitrogen Co. (Carlsbad, CA). Glycerin was purchased from DaeJung Chem Co. (Siheung, Korea). DMEM, Fetal bovine serum (FBS) were obtained from Thermo Fisher Scientific (Waltham, MA), EFO gro™ HDP was purchased from DYNE Bio Co. (Seongnam, Korea), Fibroblast cell (NIH/3 T3) was obtained from American Type Culture Collection (Manassas, VA), Hair follicle dermal papillary cell (HFDP) was purchased from Cell Engineering For Origin (Seoul, Korea).

### Synthesis and characterization of HA-PLGA conjugates

The synthesis of HA-PLGA was described in elsewhere [20]. In brief, hexamethylenediamine substituted HA (HA-HMDA) were prepared and dissolved in dimethylsulfoxide (DMSO, 30 mL) with a concentration of 1 mg/mL. PLGA (14 μmol) was dissolved in 6 mL of DMSO containing N,N′-Dicyclohexylcarbodiimide (DCC, 21 μmol) and N-Hydroxysuccinimide (NHS, 21 μmol). Then, the solution of PLGA was mixed with HA-HMDA solution, and stirred overnight. After then, the mixture was dialyzed against DI water for 3 days. The HA-PLGA was lyophilized for 3 days. And then, HA-PLGA was characterized by ^1^H nuclear magnetic resonance spectroscopy (NMR, AVANCE NEO 500, Bruker, Germany).

### Preparation and characterization of NPs encapsulating MXD or Rho B

MXD encapsulated HA-PLGA NPs were prepared by using W/O/W solvent evaporation with sonication [21]. First, 7.5 mg of MXD was dissolved in 0.5 mL of 2% acetic acid aqueous solution. The solution was added to the 1 wt% solution of HA-PLGA in of 6.25 mL of dichloromethane (DCM) containing 11.3 μL of Tween 80. And then, the mixture was emulsified by using a probe sonicator (VC 750, Sonics Inc., CT, USA) at 200 W of energy output for 20 min in an ice bath (pulse on 2.0 s, pulse off 1.0 s). Then, the prepared water-in-oil (W/O) emulsion was added to 33 mL of 1.5 wt% Poly(vinyl alcohol) (PVA) aqueous solution and the mixture was emulsified using a probe sonicator at 200 W of energy output for 20 min in an ice bath again to obtain HA-PLGA/MXD NPs. To evaporate DCM, prepared W/O/W emulsion was stirred overnight. The NPs were collected by ultracentrifugation at 12,000 rpm for 20 min. After centrifugation, the precipitated NPs were dispersed in 2 mL of DI water. Rhodamine B (Rho B) encapsulated HA-PLGA NPs were also prepared in the same manner as described above. The hydrodynamic diameter, zeta potential, and morphology of NPs were characterized by dynamic light scattering (Zetasizer Nano ZS90, Malvern Instruments, UK) and transmission electron microscope (TEM, H-7600, HITACHI, Japan). In addition, the loading capacity of the NPs is defined as the mass of the loaded drug in NPs divided by the mass of NPs and the encapsulation efficiency is defined as the loaded drug amount in the NPs over drug quantity in solutions [22]. To evaluate loading capacity and encapsulation efficiency, amount of MXD and Rho B in the supernatant of NPs solution were calculated through measurement of the absorbance at 289 nm for MXD and 555 nm for Rho B with UV-Vis spectrometer (Optizen 2120UV, Mecasys, Korea), respectively. Furthermore, to confirm the stability of MXD loaded HA-PLGA NPs, we measured the HA-PLGA NP’s size and amount of MXD in NPs after storing for 7 days in DI water.

### In vitro release of HA-PLGA/MXD NPs

To investigate in vitro release of MXD, HA-PLGA/MXD NPs were dispersed in 2 mL of phosphate buffered saline (PBS, pH 7.4) with 50 μg/mL concentration of MXD. Then, the suspension was transferred in dialysis membrane (MWCO 10,000 Da) and placed in 38 mL of PBS. The sample tube was shaken at 60 rpm in 37 °C conditions. At a predetermined time interval, 1 mL of PBS was collected and replaced by equivalent volume of fresh PBS. Then, each sample was analyzed with UV-Vis spectroscopy to quantify the amount of MXD released from HA-PLGA/MXD NPs. In addition, in vitro release test of MXD was also carried out using MXD aqueous solution and PLGA/MXD NPs in the same method as control experiment [21].

### Cell viability test

For cell viability test, fibroblasts were cultured in DMEM containing 10% FBS and 1% antibiotics and 5% CO_2_ condition. The cytotoxicity of HA-PLGA/MXD NPs against fibroblast was evaluated by cell proliferation (WST) assay using EZ-CYTOX kit (water soluble tetrazolium salt). First, fibroblasts (2.5 × 10^3^ cells/well) were seeded in a 96-well plate for 24 h. Then, various concentrations (2, 4, 10, 20, 40, 100, and 200 μg/mL) of HA-PLGA/MXD NPs were added to each well and incubated for 12 and 24 h, respectively. At the end of incubation, 10 μL of WST solution (EZ-CYTOX) was added to the cell and incubated for 2 h. The absorbance of each well at a wavelength of 450 nm was measured using a microplate reader (AMR-100, ALLSHENG, China).

### Cellular uptake test

For cellular uptake test, four dishes of hair follicle dermal papilla cells (2.5 × 10^6^ cells/dish) were cultured with EFO gro™ HDP containing 10% FBS and 1% antibiotics in 37 °C and 5% CO_2_ condition for 24 h. Then, the growth medium in all of the dishes was replaced to HA-PLGA/Rho B NPs or PLGA/Rho B NPs contained serum-free medium with 10 μM concentration of Rho B and incubated for 2 h. After that, the cells of each dish were washed with fresh PBS and 500 μL of 4% formaldehyde solution was added to each dish for cell fixation. The fixed cells were washed twice with fresh PBS and mounted with 1 mL of glycerin. And then, the cells were visualized using a fluorescence microscope (Eclipse TS100, Intensilight C-HGFI, Nikon, Japan).

### Skin permeation test

PLGA/MXD and HA-PLGA/MXD NPs with 300 μg of MXD was applied on the 3 surfaces of rat skin, respectively. The rat skin was placed between the receptor and donor chambers at 37 °C. The receptor chamber was filled with 22 mL of PBS buffer (pH 7.4). Then, at predetermined time points (4, 6, 8, 12, and 24 h), 1 mL of the sample solution was collected from a sidearm of receptor chamber and replaced by the same volume of fresh PBS. Then, each sample was analyzed with High Performance Liquid Chromatography (HPLC, e2695, Waters, USA) and UV-Vis spectrometer to measure the permeated MXD amount through the skin. The HPLC analysis was performed with a SunFire C18 column (100 Å, 5 μm, 4.6 × 250 mm, Waters, USA). The mobile phase was methanol/DI water/acetic acid (75:25:1, HPLC grade, v/v/v) and the flow rate was 1.0 mL/min. The detection wavelength was 285 nm.

### Histological analysis

Ten microliters of diluted solution of HA-PLGA/Rho B NPs and PLGA/Rho B NPs with 30 μg/mL concentration of Rho B were applied on rat skin in the same area. Then, at the predetermined time of 4, 6, 8, and 12 h, the skin tissues were harvested. The retrieved skin tissues were embedded into optimal cutting temperature (OCT) compound at − 30 °C, and cut into 10 μm thick sections using cryotome (CM1860, Leica Biosystem, Germany). The sections were fixed with the 1:1 (v/v) mixture of ethanol and acetone and washed with distilled water to remove residual OCT resins on the slide. And then, the histological tissue sections were imaged by using a fluorescence microscope. The image analysis was performed using Image J (NIH).

### Statistical analysis

Data are expressed as means ± standard deviation in a few separate experiments. Statistical analysis was carried out with the *t*-test using Prism 8 (GraphPad Software, San Diego, CA), *P* values less than 0.05 were considered statistically significant.

## Results

### Synthesis of HA-PLGA

**Fig. 1:**
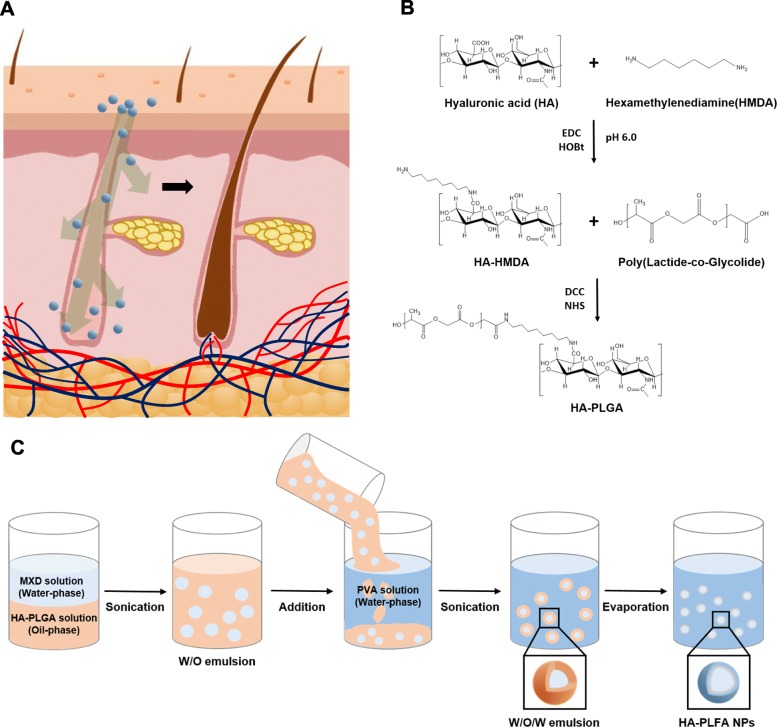
Schematic representation for **a** transdermal delivery of HA-PLGA NPs, **b** synthesis of poly(lactic-*co*-glycolic acid) grafted hyaluronic acid (HA-PLGA) and **c** W/O/W emulsification of HA-PLGA

**Fig. 2:**
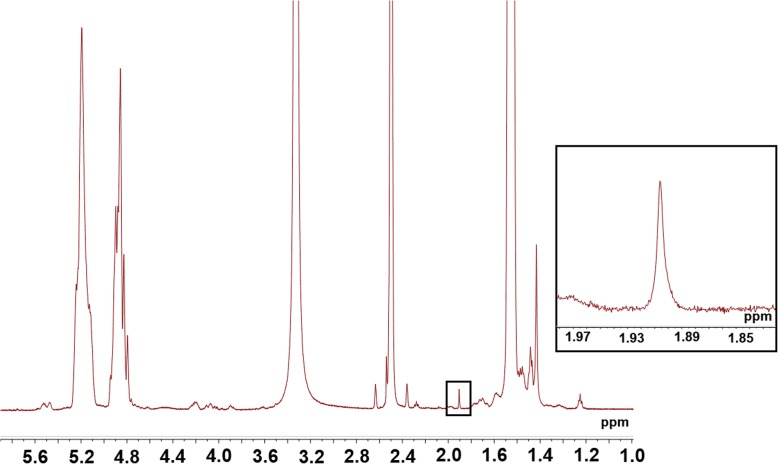
^1^H nuclear magnetic resonance (NMR, DMSO-d_6_) spectrum of HA-PLGA. The methyl peak of acetamido group in HA at δ = 1.9 ppm (inset)

HA-HMDA with 30 mol% of HMDA was used for the conjugation with PLGA. Then, HA-PLGA was synthesized by conjugation between the amine group of HA-HMDA and the carboxyl end group of PLGA in DMSO (Fig. 1b). After purification and lyophilization, HA-PLGA was dissolved in DMSO-d_6_ with concentration of 10 mg/mL and characterized by ^1^H NMR. The characteristic peaks of methyl resonance of acetamido moiety of HA at δ = 1.9 ppm and the methyl resonance of LA of PLGA at δ = 1.5 ppm were shown in NMR spectrum (Fig. 2). From each characteristic peak, we could assess grafting ratio of PLGA for HA was 4.4%.

### Characterization of PLGA/MXD and HA-PLGA/MXD NPs

After preparing the NPs, we carried out the characterization of PLGA/MXD NPs and HA-PLGA/MXD NPs. The loading efficiency of MXD in NPs could be calculated by measuring the absorbance of supernatant. The drug loading and encapsulation efficiency of PLGA/MXD NPs and HA-PLGA/MXD NPs were summarized in Table 1. More than 40% of MXD was encapsulated in both of PLGA NPs and HA-PLGA NPs and it was shown that MXD was more loaded in NPs conjugated with HA. These results and the transdermal characteristic of HA could show a synergistic effect and enable more efficient delivery of MXD. Therefore, we expected HA-PLGA NPs would be a good candidate of MXD carrier for alopecia treatment.

**Table 1:** Characterization PLGA/MXD and HA-PLGA/MXD NPs

	Mean volume diameter (nm)	PDI	Minoxidil content in nanoparticles (%)	Encapsulation efficiency (%)
PLGA/MXD	159 ± 11.8	0.074	4.66	40.70
HA-PLGA/MXD	243 ± 44.5	0.182	6.24	55.48

**Fig. 3:**
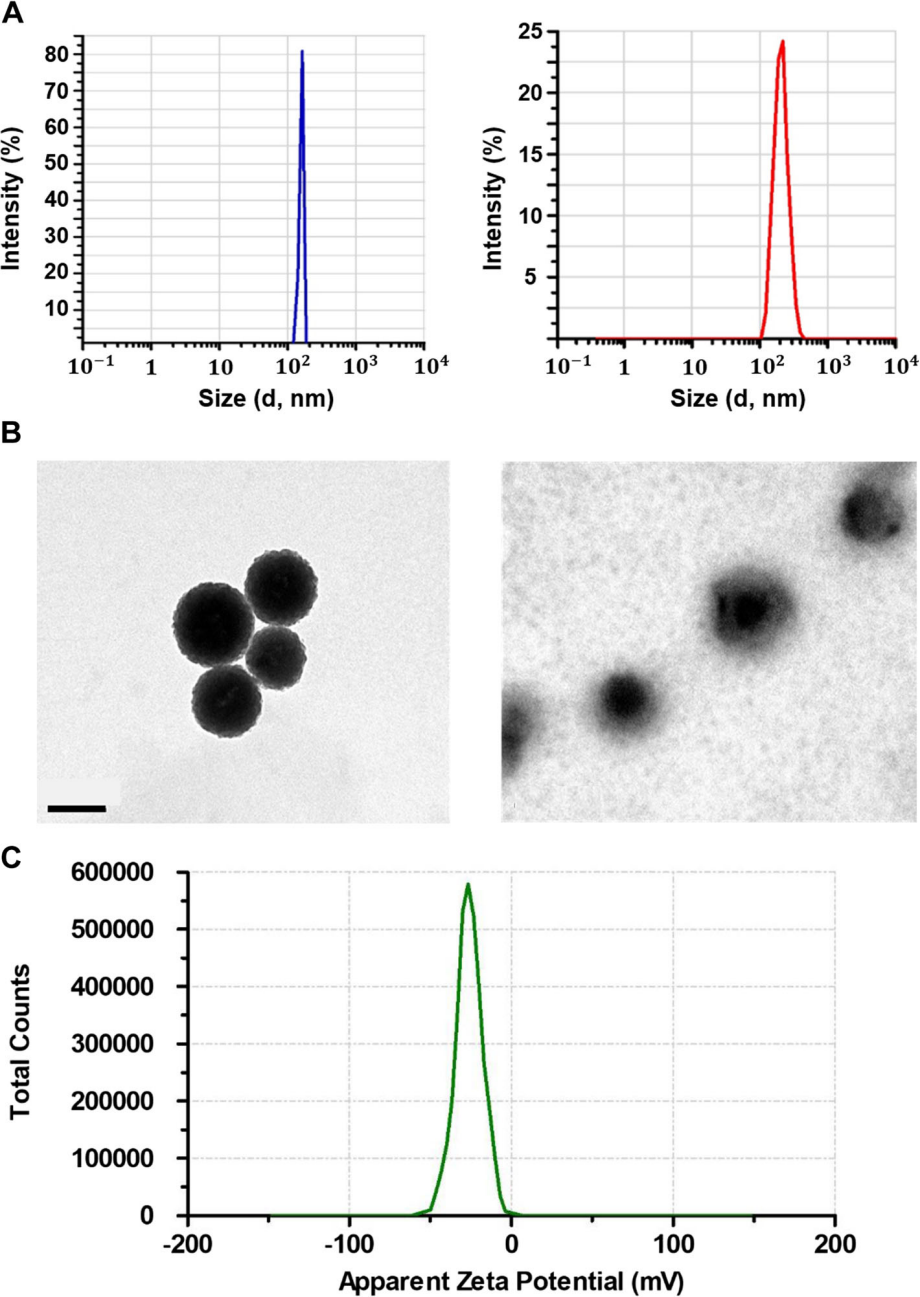
**a** The size distribution of PLGA/MXD NPs (blue) and HA-PLGA/MXD NPs (red) measured by DLS. **b** TEM images of PLGA/MXD NPs (left) and HA-PLGA/MXD NPs (right). Scale bar indicates 200 nm. **c** Zeta potential of HA-PLGA/MXD NPs

Hydrodynamic diameters and zeta potential of each NP encapsulating MXD were measured by DLS (Fig. 3a, c). The mean hydrodynamic diameter of PLGA/MXD NPs was 159 ± 11.8 nm (PDI: 0.074) and that of HA-PLGA/MXD NPs was 243 ± 44.5 nm (PDI: 0.182). Although PDI was increased with the conjugation of HA, the size distribution of HA-PLGA NPs was still narrow and located in appropriate size, which could be expected to be delivered to hair follicles. As shown in Fig. 3c, NPs had a negative surface charge ranging from − 43.2 to − 0.4 mV after conjugation with negatively charged HA. In addition, the TEM image revealed that both of PLGA/MXD and HA-PLGA/MXD NPs had the spherical structure with uniform size. Especially, HA-PLGA NPs were shown core-shell structure composed by hydrophobic PLGA core and hydrophilic HA shell (Fig. 3b). We could confirm that the size of NPs and amount of MXD in NPs were maintained stably for 7 days in DI water.

### Drug release profiles of MXD-loaded HA-PLGA NPs

**Fig. 4:**
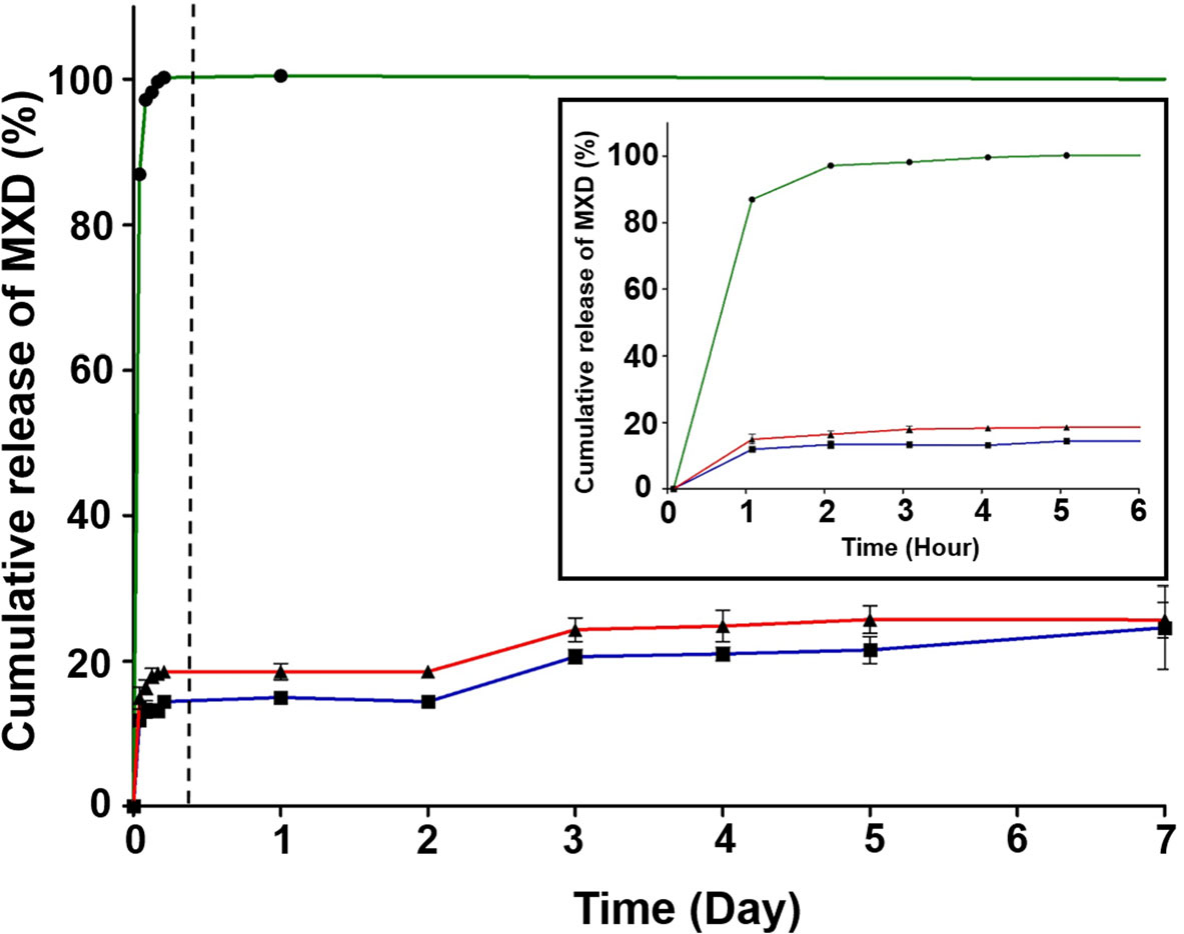
Cumulative release profile of MXD aqueous solution (●), PLGA/MXD NPs (■) and HA-PLGA/MXD NPs (▲) for 7 days. Extended cumulative release profile for 6 h (inset)

In vitro release test of MXD encapsulated in HA-PLGA NPs was carried out with dialysis membrane (MWCO 10,000 Da) for 7 days. As a control, the PLGA/MXD NPs and MXD aqueous solutions were also performed in the same condition. Using collected 1 mL of PBS at a predetermined time, the released amount of MXD could be measured by UV-Vis absorbance at 285 nm. Figure 4 shows the cumulative release of MXD from PLGA NPs and HA-PLGA NPs in PBS at 37 °C. MXD was released steadily from both PLGA NPs (24%) and HA-PLGA NPs (25%).

### In vitro cellular uptake of NPs and cytotoxicity test

**Fig. 5:**
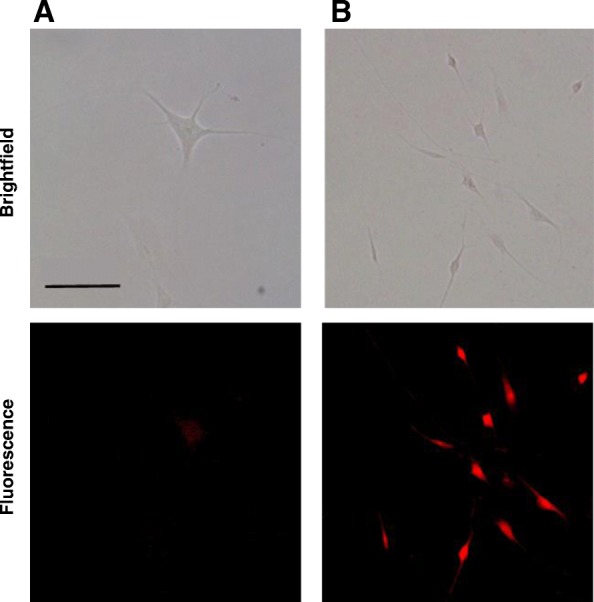
Confocal microscopic images of uptake and accumulations of **a** PLGA/Rho B NPs and **b** HA-PLGA/Rho B NPs in dermal papillary cells. Scale bar indicates 100 nm

**Fig. 6:**
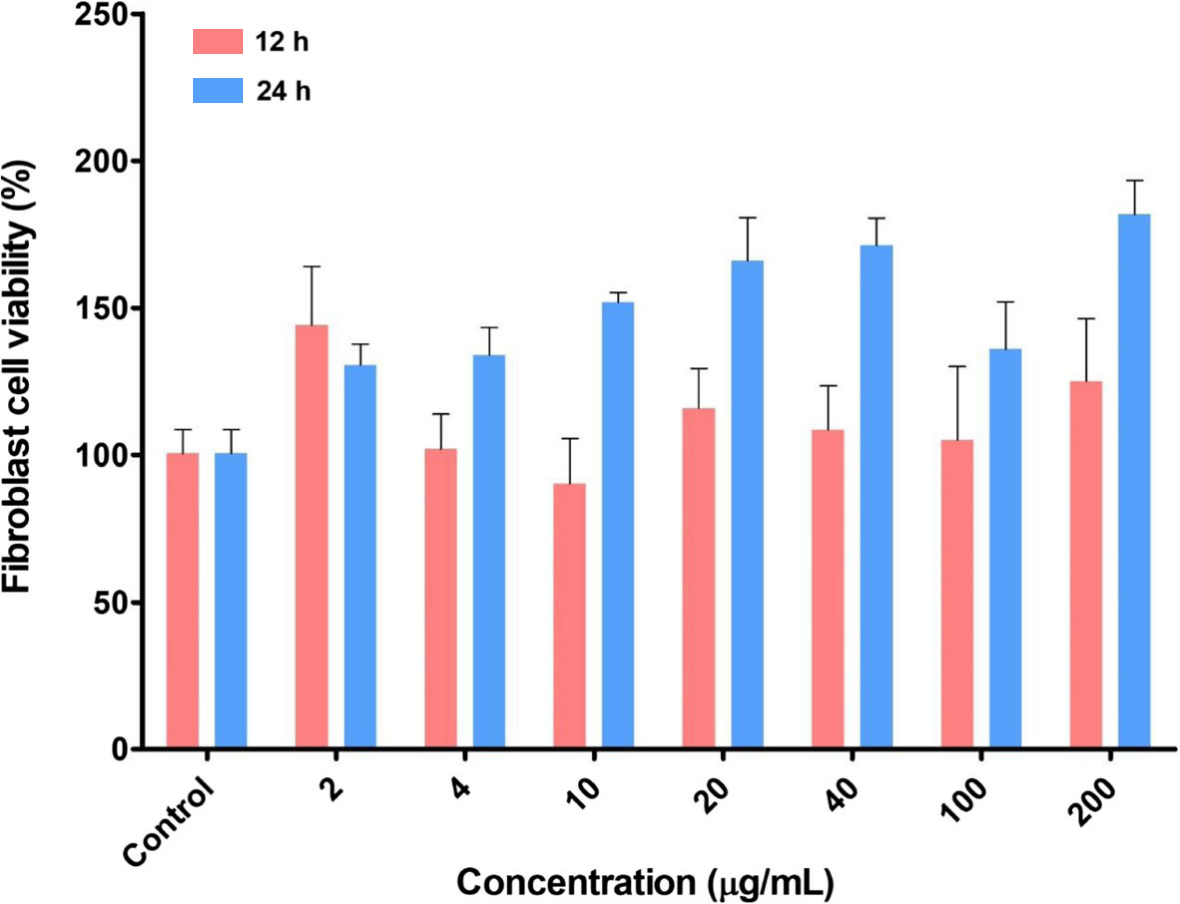
In vitro cytotoxicity of HA-PLGA/MXD NPs in Fibroblast cells evaluated by WST assay for 24 h

To investigate cellular interaction of NPs, we confirmed the uptake of Rho B loaded NPs by hair follicle dermal papillary cells which regulate hair follicle development and growth in vitro using fluorescence microscopy. As the results, we could confirm that the HA-PLGA/Rho B NPs treated cells showed strong fluorescence while PLGA/Rho B NPs showed little fluorescence in the cells (Fig. 5). In addition, cell viability of HA-PLGA/MXD NPs was investigated using mouse fibroblast cells (Fig. 6). When we incubated NPs to the cells with different concentrations of MXD from 2 μg/mL to 200 μg/mL and incubation time of 12 h and 24 h, respectively, there was no significant cytotoxicity of MXD encapsulated NPs to the cells.

### Skin permeation of MXD encapsulated NPs

**Fig. 7:**
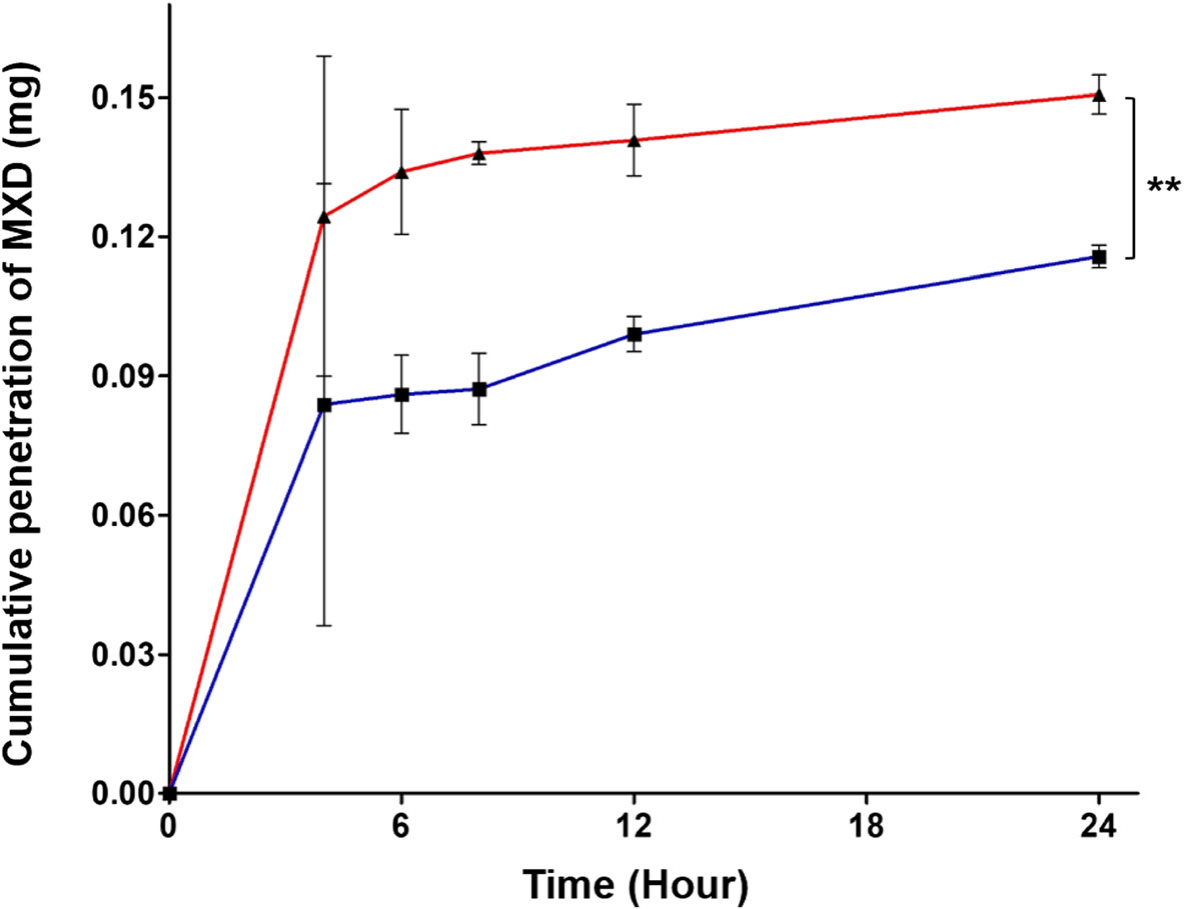
Skin penetration of MXD from PLGA/MXD (■) and HA-PLGA/MXD NPs (▲) investigated by using a static Franz diffusion cell (mean ± SD, *n* = 3, **p < 0.01, *t*-test)

**Fig. 8:**
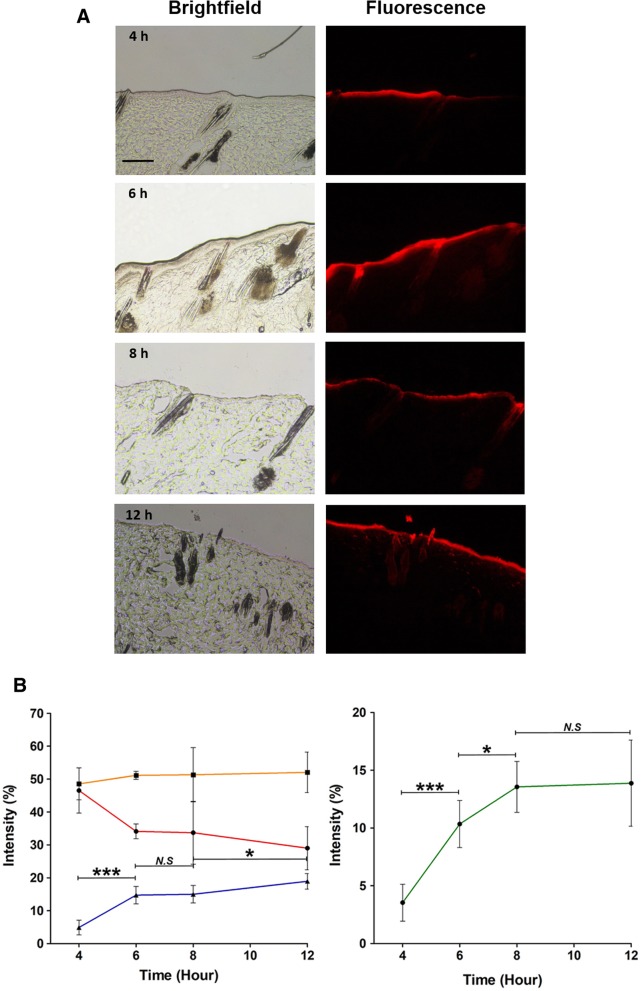
**a** Fluorescence microscopic images of histological section of rat skin at 4, 6, 8 and 12 h after topical application of Rho B encapsulated HA-PLGA NPs. Scale bar indicates 100 μm. **b** Quantitative analysis of fluorescence intensity at each skin layer: stratum corneum (●), epidermis (■), dermis and follicle cells (▲) and only follicle cells (♦). (mean ± SD, *n* = 5, ***p < 0.001, *t*-test)

Based on in vitro results, we investigated the efficiency and dynamics of transdermal permeation using a static Franz diffusion cell and histological examinations. At first, the skin penetration of PLGA NPs and HA-PLGA NPs with 300 μg of MXD was investigated for 24 h. The content of MXD in the NPs penetrating into skin tissue increased with time in the receptor of Franz diffusion cell (Fig. 7). After 4 h, 124.4 ± 0.034 μg of MXD was measured in HA-PLGA NP samples beside 83.8 ± 0.048 μg of MXD in PLGA NP samples and after 24 h, 150.6 ± 0.004 μg in HA-PLGA NP samples and 115.7 ± 0.002 μg in PLGA NP samples were measured. From the results, we confirm that there was a significant difference in the cumulative amount of MXD between both of the samples with statistical analysis. Since the previous release profile test did not show a significant difference between HA-PLGA NPs and PLGA NPs, the results of this experiment were affected by permeability more than release rate. As the results, we can confirm that HA-PLGA NPs were more permeable than PLGA NPs and more efficiency in transdermal delivery. In addition, for the histological analysis, HA-PLGA/Rho B NPs were topically applied on rat skin tissue. Confocal images (Fig. 8a) and variation of fluorescence intensity in each region (Fig. 8b) of tissue sections harvested at a predetermined time after topical administration showed a time-dependent increase of HA-PLGA/Rho B NPs in the hair follicles. At 4 h, 95% of total fluorescence was measured in stratum corneum and epidermis and it meant that HA-PLGA/Rho B NPs were still only on the surface of the skin. But after 6 h, while the intensity ratio of stratum corneum decreased, the other region’s were constant, but the follicle cell’s was increased.

## Discussion

Transdermal drug delivery is the most attractive candidate for replacing needle-injection. However, there are some limitations, such as low delivery efficiency and bioavailability. In case of the treatment of alopecia, MXD is widely used, but using MXD also has disadvantages when it is applied on the skin. To increase the delivery efficiency of MXD, we designed novel transdermal carriers in forms of nanoparticles. To use effectively each material with good properties in the delivery of MXD, we used an emulsification method through solvent evaporation [21, 23, 24]. Emulsion method can facilitate more efficient storage, improve stability of hydrophobic drugs, and reduce the size of particles as drug delivery carrier [18]. Therefore, we prepared the HA-PLGA NPs using W/O/W emulsion method which has been widely investigated in drug delivery system [18].

At first, in the HA-PLGA synthesis, we could confirm the successful synthesis of HA-PLGA from the characteristic peaks in NMR spectrum and 4.4% of substitution of PLGA for HA. Then, after characterization of HA-PLGA NPs, although PDI was increased with the conjugation of HA, the size distribution of HA-PLGA NPs was still narrow and located in appropriate size for the hair follicle permeation, which could be expected to be delivered to hair follicles. In addition, HA-PLGA NPs were shown core-shell structure composed by hydrophobic PLGA core and hydrophilic HA shell by TEM analysis.

In vitro release and cellular uptake test, the HA-PLGA NPs showed the controlled release of MXD and high efficiency of cell uptake compared to PLGA NPs. The mechanism of cellular uptake is HA receptor-mediated endocytosis by the highly expressed CD 44 which is well-known to HA receptor on dermal papillary cells [13]. Therefore, the biocompatible HA conjugated NPs can be more utilized in the treatment of alopecia.

Through the permeation test, we could confirm that HA-PLGA NPs gradually penetrated through the hair follicles. 8 h later, the fluorescence intensity in the epidermis and follicle cells were almost constant, but the total intensity in stratum corneum was decreased continuously and the those in the dermis and follicle cells were increased. Thus, these results suggest that HA-PLGA NPs delivered to the follicle cells were dispersed to the dermis and took 8 h to approach the surrounding tissue.

Taken together, HA conjugated NPs could show a synergistic effect and enable more efficient delivery of MXD by their excellent skin permeability characteristics. Therefore, we expected HA-PLGA NPs would be a good candidate of MXD carrier for alopecia treatment.

## Conclusion

In this work, we prepared MXD-encapsulated HA-PLGA NPs using W/O/W solvent evaporation method and confirmed the biocompatibility in cell viability test and the ability of HA-PLGA NPs to delivery to follicle cells in cellular uptake test and skin permeation test. From the characterization of HA-PLGA NPs, we could confirm higher efficiency of transdermal delivery that HA-PLGA NPs had not only suitable hydrodynamic diameter, but also more drug loading efficiency than PLGA NPs. In addition, a sufficient amount of MXD was uptaken into cells without cytotoxicity and HA-PLGA NPs were successfully delivered to hair follicle cells. With these results, HA-PLGA NPs are suitable in transdermal delivery of MXD for alopecia treatment and can be exploited to develop an efficient and effective platform for the treatment in various other diseases.

## Data Availability

Please contact author for data request.

## Abbreviations

HA: Hyaluronic Acid
MXD: Minoxidil
NPs: Nanoparticles
PLGA: Poly(Lactide-*co*-Glycolide)
